# Polydopamine Functionalization: A Smart and Efficient Way to Improve Host Responses to e-PTFE Implants

**DOI:** 10.3389/fchem.2019.00482

**Published:** 2019-07-09

**Authors:** Isabelle Talon, Anne Schneider, Vincent Ball, Joseph Hemmerle

**Affiliations:** ^1^Service de Chirurgie Pédiatrique, Hôpitaux Universitaires de Strasbourg, Strasbourg, France; ^2^Institut National de la Santé et de la Recherche Médicale, UMR_S 1121, Strasbourg, France; ^3^Faculté de Chirurgie Dentaire, Université de Strasbourg, Strasbourg, France

**Keywords:** e-PTFE, congenital diaphragmatic hernia, prosthesis, side specific functionlization, polydopamine, water/air interface

## Abstract

Among the different materials used as protheses for the treatment of Congenital Diaphragmatic Hernia, expanded polytetrafluoroethylene (e-PTFE) plays a leading role owing to its mechanical properties as explained in the first part of this review. However, this material is poorly cell adhesive, which is expected for its contact on the abdominal face, but should display specific tissue adhesion on its thoracic exposed faced. A side specific functionalization method is hence required. The deposition of a nanosized polydopamine film on PTFE is known to be possible but immersion of the e-PTFE membrane in an aerated dopamine solution leads to a functionalization not only on both faces of the membrane but also in its porous volume. The fact that polydopamine also forms at the water/air interface has allowed to transfer a polydopamine film on only one face of the e-PTFE membrane. The deposition method and applications of such Janus like membranes are reviewed in the second part of the review.

## Introduction

Congenital Diaphragmatic Hernia (CDH) is a congenital malformation defined as a diaphragmatic defect with intrathoracic herniation of abdominal viscera and pulmonary hypoplasia. It occurs in 1:2,000 to 1:3,000 births (McGivern et al., 2015). Despite pre-natal diagnosis, development of fetal surgery, and codified intensive care, mortality rate after birth is still high. Diaphragmatic defect size directly impacts survival rate: the bigger the defect, the higher is the mortality rate. Thus, CDH are classified according the side of the defect (right or left or bilateral) and the size of the defect: from a small one (type A) to a total left or right diaphragmatic agenesis (type D).

Treatment is based on intensive resuscitation and surgical repair of the defect. In case of large defects, surgery is based on the use of a prothesis. Finally, a prosthesis is needed for more than half of the cases. The challenge for these prostheses is related to the fact that the diaphragm is a unique and essential muscle. It is the main ventilatory muscle that will grow by a factor of four between the neonatal period and the adolescence (Schneider et al., 2017a). It is subject to important pressure gradients and also plays a role in the spinal balance: a lack of compliance of the diaphragm is responsible for spinal static disorders (Steigman et al., 2010). It is known that respiratory complications, recurrences of hernias and spinal disorders are more common in children whose diaphragmatic defect was repaired with a prosthetic membrane (Tsai et al., 2012; Jancelewicz et al., 2013; Jawaid et al., 2013). Therefore, the prosthesis has to be stretchable, to follow the growth of the child, to withstand the pressure gradients and to be sufficiently compliant to limit the constraints imposed on the spine. In this review article we will first describe the materials used to seal CDH and in a second section how polydopamine functionalization of a particularly well-suited coating to allow a side specific functionalization of the most used material, namely expanded Polytetrafluoroethylene.

## Materials Used as Diaphragmatic Prosthesis

The nowadays most commonly used diaphragmatic prosthesis is expanded polytetrafluoroethylene (Gore-Tex®, W.L. Gore and Associates, Flasgstaff, AZ) usually shorten in expanded polytetrafluoroethylene (e-PTFE). This biomaterial is used in more than 80% of diaphragmatic replacements (Eastwood et al., 2018). e-PTFE is an inert porous and resistant material that it is suitable for surgery. It's textured surface on one side allows host tissue penetration as shown by Schneider et al. (2017b). Nevertheless, as it lakes elasticity, it is not the ideal mesh for the repair of diaphragmatic defects. In this respect, it must be emphasized that this type of prosthesis (ePTFE) is associated with recurrence rates up to 50%. Improvement strategies have been proposed to try to reduce the rate of recurrence, for example, the modeling of e-PTFE plates in order to obtain a cone or dome shape or the realization of a double fixation of the prosthesis (Loff et al., 2005).

Composite prostheses have also emerged, such as the PTFE-Marlex® (CR Bard, Murray Hill, NJ) composite. This particular PTFE is thought to prevent adhesion at the face oriented toward the abdomen and to promote tissue integration at the opposite side that is composed of monofilament microporous polypropylene. A retrospective monocentric study showed a recurrence rate of around 4% in a series of 28 patients over a 4 year follow-up after use of this last biomaterial. Unfortunately, there was a significant splenic injury rate (17%) requiring splenectomy (Riehle et al., 2007).

Edwards et al. reported two successful cases where a composite polyester/collagen mesh was used (Wang et al., 2015). This prosthesis has been used for decades for parietal hernia in adults, but no report was found for CDH (Parietex®, Covodoen, Sofradim, France). It must be pointed out that one of the two cases was an adult girl without the challenge of growing and the second case had a follow up of only 8 months postoperatively and a lake of information about spinal static state and growth.

On an other hand, absorbable alternatives are increasingly used, as Surgisis® (SIS®, Cook Biotech Inc., Cook, Deutchland GmbH, Mönchengladbach) which is a collagen matrix derived from decellularized porcine intestinal submucosa, grafted with growth factors. This biomaterial, however, leads to major inflammatory reactions, due to the presence of pig DNA (due to not completely decellularized matrix), with still unknown potential consequences for humans (Grethel et al., 2006). When this biomaterial is used alone, recurrences occur earlier, possibly due to a too rapid degradation of the material in comparison with slow muscle regeneration. A recent meta-analysis concluded that there is no significant difference in terms of recurrence between Surgisis® and e-PTFE (Romao et al., 2012).

AlloDerm® (Lifecell Corporation, Branchburg, NJ), another absorbable material, made of decellularized extracellular matrix derived from the dermis of human cadavers, is used less often for the discussed indication, because the product leads to high recidivism rates (40%) and especially to second recurrence (Grethel et al., 2006; Laituri et al., 2010). Indeed, this decellularized matrix offers a basement membrane framework for host cell colonization. But this absorbable meshes have to withstand the strength of the repair until host colonization of the biomaterial. This appears to be the weakness of that material in the indication of diaphragm repair. It must be mentioned that some researchers attempted to use chemical cross-linking to avoid a too fast degradation of the implanted mesh (Badylak, 2004).

Urita et al. evaluated the efficacy of diaphragmatic hernia repair in a rat model using a poly-lactic-co-glycolic acid (PLGA) mesh–collagen sponge hybrid scaffold (Urita et al., 2008). They compared three different grafts: a PLGA mesh (*n* = 7), a PLGA mesh–collagen sponge hybrid scaffold (*n* = 7), and a PLGA mesh–collagen sponge hybrid scaffold seeded with bone marrow-derived mesenchymal stem cells (MSCs) (*n* = 10). The neo-diaphragm was significantly thicker in the case of PLGA mesh-collagen sponge with no significant difference with scaffolds seeded with MSCs. On the other hand, the PLGA mesh–collagen sponge hybrid scaffold favored autologous *in situ* tissue regeneration in the diaphragm, suggesting its potential application for diaphragmatic repair in place of other prosthetic patches.

Permacol TM® (Covidien, AG) is a collagenous matrix derived from decellularized porcine dermis with HDMI (hexamethylene diisocyanate) cross-linking that resists breakdown from collagenase. Crosslinking confers an increase in tensile strength to the material. This biomaterial was implanted in eight patients, without occurrence of recurrence on a follow-up of 20 months. But this crosslinked matrix did not demonstrate significant difference compared to e-PTFE (Mitchell et al., 2008). Recently, Filisetti et al. reported their own experience and a literature review on Permacol® in pediatric surgery (Filisetti et al., 2016). One can note that there are no details on the indication of Permacol® in diaphragmatic hernia apart from four records concerning, for two of them, late discoveries of the pathology and, for two others, secondary interventions after Gore-Tex failures. For the four cases, they did not report any complication or requirement of removal of the implant patch. As things stand now, these data are clearly insufficient to draw conclusions about this biomaterial in the management of large defect neonate hernia.

More recently, Deprest et al. compared a radial pore oriented acellular collagen scaffold to two clinical used surgical meshes (Gore-Tex and Surgisis) in a surgically induced rabbit diaphragmatic tissue defect model (Eastwood et al., 2018). They found no differences in terms of hernia recurrence but a compliance closer to that of the native diaphragm for their scaffold compared to Gore-Tex. The foreign body reaction seems to be less important with their scaffold than with Gore-Tex but with a majority of implants showing calcification foci, elements that were already reported concerning collagen materials (Nuininga et al., 2004) possibly due to cell debris or elastin or to a differentiation of macrophages into cells of osteoblastic phenotype (Mosala Nezhad et al., 2016).

Lastly, regenerative medicine is an option that is being studied more and more in the context of diaphragmatic hernias, with many experimental works ongoing (De Coppi and Deprest, 2017) including a very recent one combining the use of a novel bio-3D printer method to generate large scaffold-free tissue patches composed of human cells. The resulting large tissue constructs provide high elasticity and strength. Cellular patches were transplanted in rats with surgically created diaphragmatic defects (Zhang et al., 2018).

## Functionalization of e-PTFE With Polydopamine

Owing to the previous description, it appears that e-PTFE based materials are by far the best prosthesis candidates for CDH treatment based on their mechanical properties. However, their chemical inertness makes them poor candidates for tissue adhesion of the thoracic face even if they appear optimal for the abdominal face. The chemical functionalization of materials remained material specific for many years with adapted chemistries for each great materials classes (metals, oxides, polymers), Teflon remaining almost impossible to functionalize with a selected class of molecules. Twelve years ago, inspired by findings showing that the high abundance of L-Dopa and L-Lysine in the proteins present in the mussel byssus was responsible for the strong under water adhesion of mussels to vast repertoire of materials (Lee et al., 2011), it was shown that dopamine allows producing conformal coatings on almost all known materials. Indeed, the use of dopamine in oxidizing conditions allows depositing a conformal coating on all known kinds of materials including PTFE (Lee et al., 2007; Ryu et al., 2018). The obtained material is called polydopamine (PDA) and is structurally and functionally very close to eumelanin, the natural photoprotectant of the skin and a natural pigment of the eye and the hairs (Meredith and Sarna, 2006).

Since then, PDA has been extensively used to modify the surface of biomaterials and to elicit specific cell response (Lynge et al., 2010; Liu et al., 2014, 2015). It is hence an excellent candidate to functionalize e-PTFE prostheses with a biocompatible anchoring layer.

**Figure 1:**
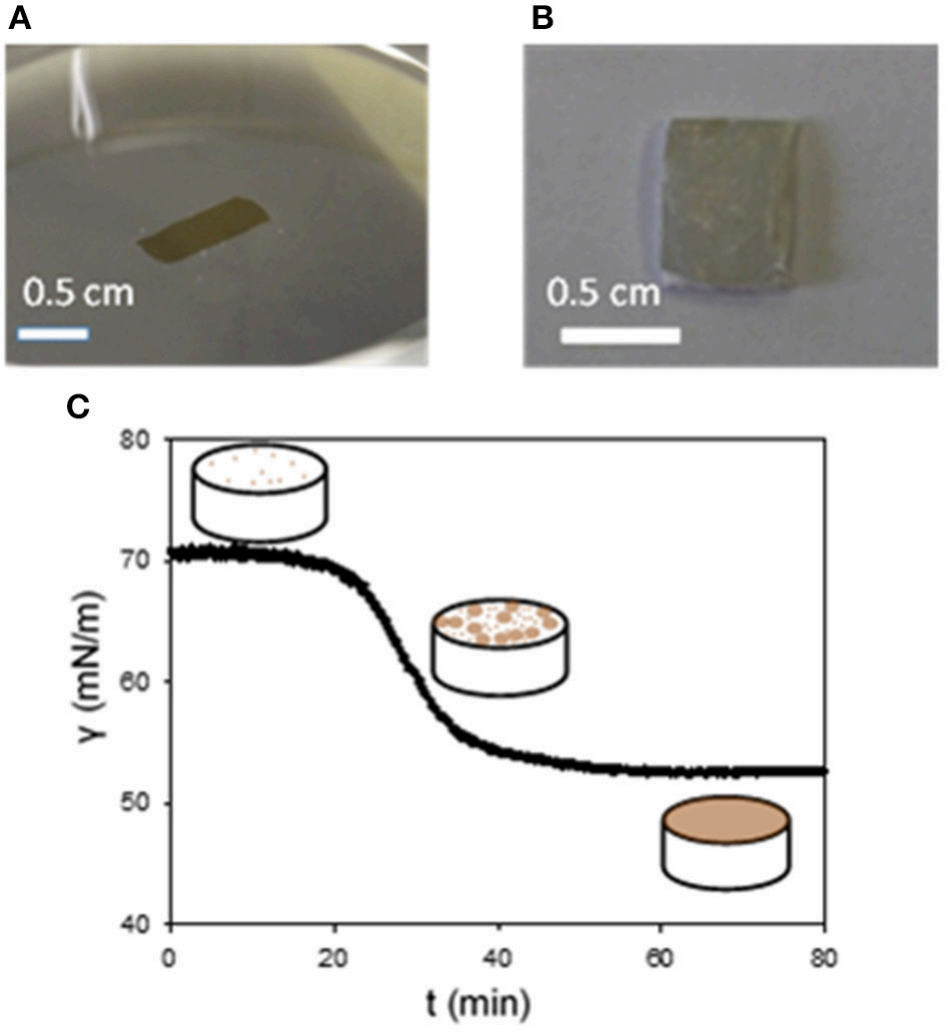
Pictures the surface of a PDA film at the water/air interface **(A)** and after its transfer on a e-PTFE membrane **(B)**. **(C)** Change of the surface tension vs. time in a pendant drop configuration. The dopamine concentration in the Tris buffer (10 mM, pH = 8.5) was of 2 mg.mL^−1^. The scheme depicts the progressive coverage of the air/water interface with PDA. Reproduced from Ponzio et al. (2014) with authorization.

In the particular case of e-PTFE, the immersion of the membrane causes not only the deposition of PDA on both faces of the membranes, but also through its porous volume. Owing to the materials requirement of e-PTFE as a biomaterial for the treatment of CDH, it is required to functionalize exclusively the thoracic face of the membrane keeping the abdominal side non-adhesive as already explained. Fortunately, PDA can also be deposited at the solution/air interface (Hong et al., 2014; Yang et al., 2014) opening the possibility to transfer PDA films in the same manner as amphiphiles using the Langmuir-Schaeffer (Langmuir and Schaeffer, 1936) deposition method (Ponzio et al., 2014; Ponzio and Ball, 2016). The deposition of PDA at the solution/air interface from the highly soluble dopamine shows that the oxidation product of dopamine is amphiphilic. The deposition occurs only after an initial lag phase, which does not occur upon PDA deposition at the solid/solution interface, as shown by means of tensiometry (Figure 1). Note that the same kind of transfer allows also the transfer of polyaniline films from the solution/air interface to solid substrates.

**Figure 2:**
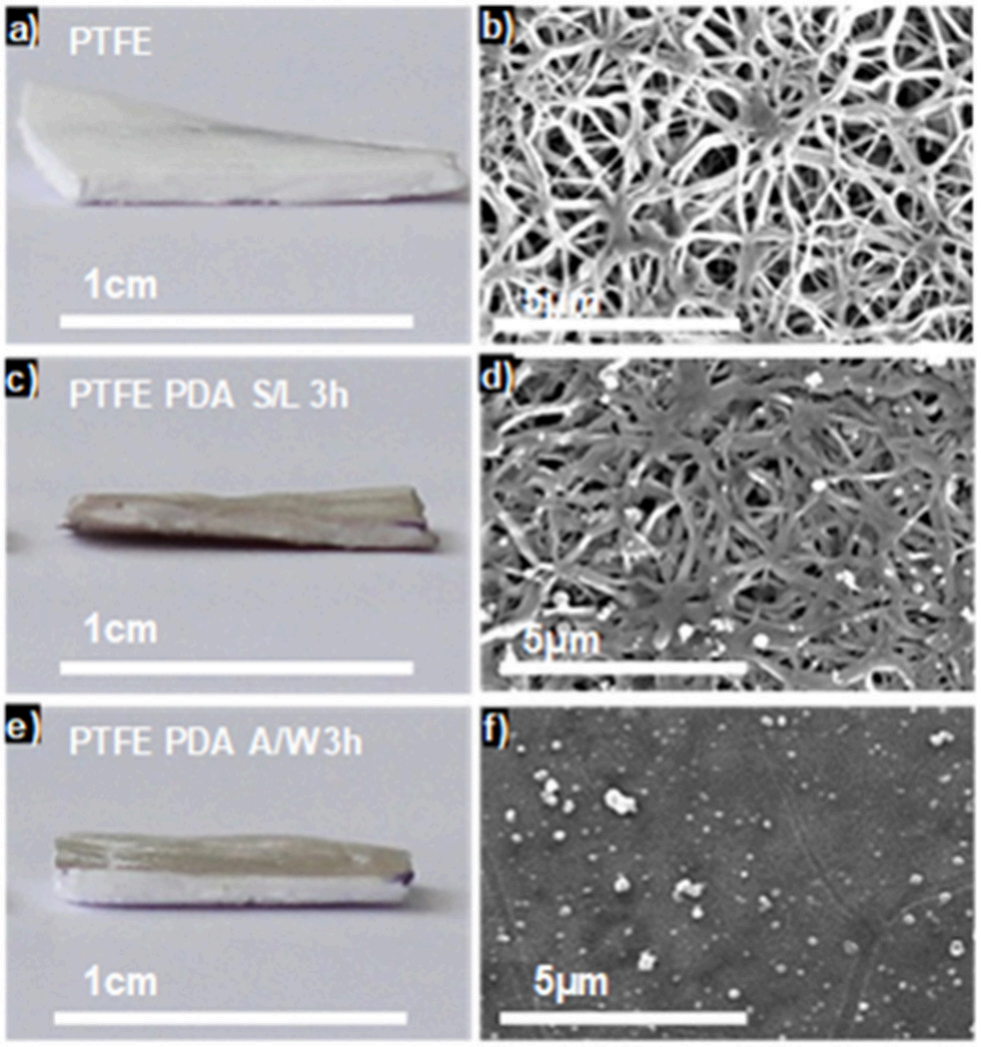
Optical pictures **(a,c,e)** and top view SEM images **(b,d,f)** of raw e-PTFE **(a,b)**, PTFE-PDA obtained by immersing the membrane in a dopamine solution (2 mg.mL^−1^ at pH = 8.5) after 3 h of reaction **(c,d)** and e-PTFE-PDA obtained after Langmuir-Schaeffer deposition from the solution/air interface after 3 h of reaction **(e,f)**. Reproduced from Ponzio et al. (2014) with authorization.

Using this finding, we showed that combining PDA chemistry and transfer of the e-PTFE membrane trough the water/air interface, previously covered by a PDA thin film, allowed the selective functionalization of only one face of the e-PTFE membrane (Ponzio et al., 2014; Figure 2).

The coatings obtained directly through deposition at the solid/water interface and those deposited through Langmuir-Schaefer transfer from the water/air interface were characterized by means of X-ray photoelectron spectroscopy (XPS) and atomic force microscopy (AFM) concerning their chemical composition and surface morphology, respectively. In particular the N/C ratio of the PDA films deposited directly on e-PTFE and those transferred from the water/air interface on e-PTFE was identical and equal to 0.12 ± 0.1 as expected for PDA (the N/C ratio in dopamine is 0.125 and no carbon nor nitrogen should be lost in the absence of degradation) (Ponzio et al., 2014, and Table S1 of the Supporting Information in this reference). The PDA films deposited on e-PTFE and those transferred thereon displayed similar surface morphology as shown by AFM (see Figure S11 in Ponzio et al., 2014).

Owing to the ability of PDA to bind proteins through a combination of covalent and non-covalent interactions (Bernsmann et al., 2010), those asymmetrically functionalized ePFTE membranes could be ideal as membranes for the surgical repair of diaphragm defects.

Tissular integration of a biomaterial that separates the thorax from the abdomen is not trivial, as many biomaterials used for tissue engineering applications lack cell-adhesiveness. For this reason, e-PTFE has to be chemically activated. To verify the relevance of e-PTFE functionalization with PDA, we assessed the biological responses of a clinically used e-PTFE biomaterial treated with PDA in the two different manners: one impregnated with PDA and the other coated with a one side PDA film.

**Figure 3:**
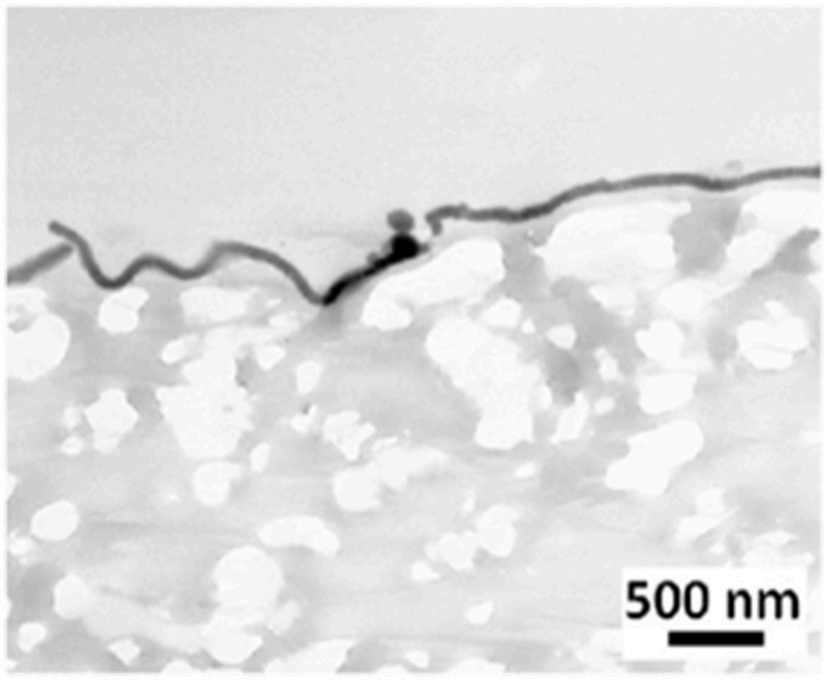
TEM micrograph showing a thin PDA film on a e-PTFE biomaterial surface. Scale bar = 500 nm. Reproduced from Talon et al. (2019) with authorization.

Treatment of a biomaterial by dipping it into a dopamine solution paves the way for numerous secondary functionalization methods. Despite its evident interest in the medical field, the dipping of a porous implant material, as e-PTFE, in a PDA solution produces a biomaterial sheet with the same properties on each side. With the goal of modifying exclusively one side of the porous e-PTFE material, we used the Janus like e-PTFE membranes, that is to say a PDA film deposited at one side only by means of Langmuir-Schaeffer transfer from the water/air interface (Figure 3). This original approach is of high concern in the case of CDH implants, where the expected host responses have to be different at each side: the surface facing the abdominal cavity should prevent tissue adhesions, thus avoiding bowel occlusion.

**Figure 4:**
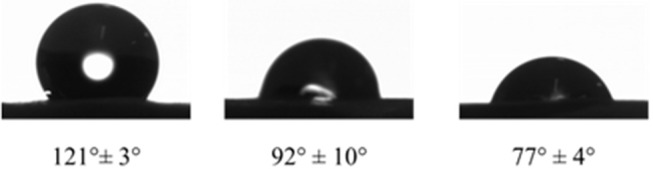
Water contact angles of raw e-PTFE (121 ± 3°), PDA-impregnated e-PTFE (92 ± 10°), and PDA-coated e-PTFE (77 ± 4°), at room temperature on the rough side of the biomaterial. Reproduced from Talon et al. (2019) with authorization.

Measurements of static contact angles show that the PDA-coated e-PTFE is more hydrophilic than the PDA-impregnated e-PTFE material (Figure 4). Both functionalization methods do not induce cell mortality, but the PDA-impregnated ePTFE produces more TNFα (Talon et al., 2019). This can be explained by the fact that this last e-PTFE contains probably more PDA particles due to the porosity of e-PTFE. We can assume that PDA does not affect cell viability and favors cell development.

**Figure 5:**
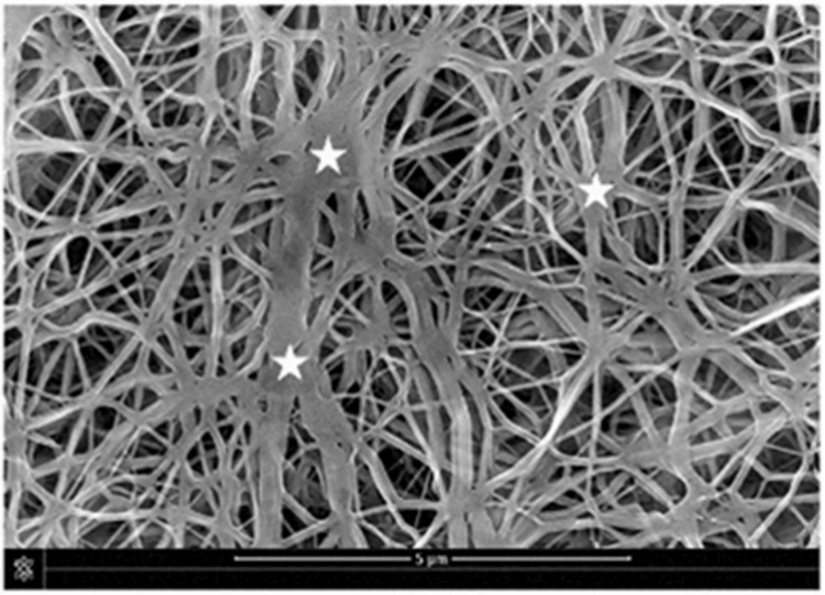
SEM micrograph disclosing the fibrillary structure of the e-PTFE material. One can note the distribution of nodes (stars) within the fibrillar PTFE network. Scale bar = 5 μm. Reproduced from Talon et al. (2019) with authorization.

**Figure 6:**
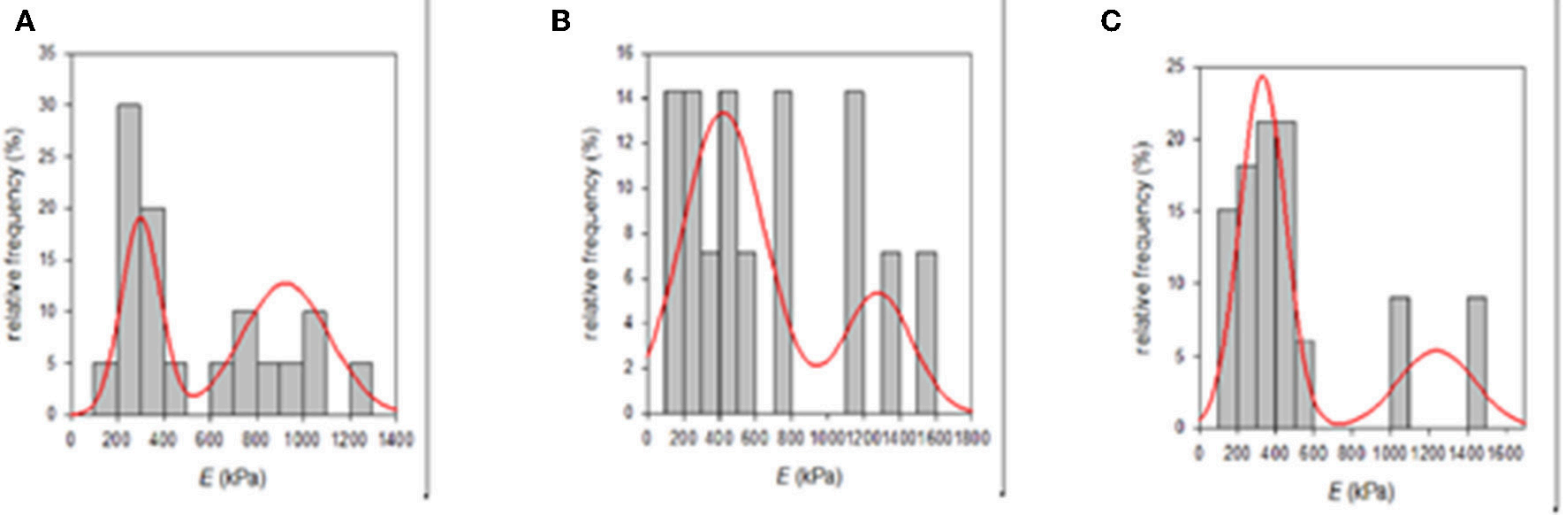
**(A)** Histogram of the Young's moduli of the raw e-PTFE raw material measured by AFM (*n* = 20). It suggests a bimodal distribution of the stiffness mirroring the heterogeneity of the material. **(B)** Histogram of the Young's moduli of PDA-impregnated e-PTFE measured by AFM (*n* = 14). It suggests a bimodal distribution of the stiffness mirroring the heterogeneity of the biomaterial. **(C)** Histogram of the Young's moduli of PDA-coated e-PTFE measured by AFM (*n* = 33). It also suggests a bimodal distribution of the stiffness mirroring the heterogeneity of the biomaterial. Reproduced from Talon et al. (2019) with authorization.

It is well-known that the mechanical properties of a substrate are able to influence single and collective cell behaviors. For this reason, we investigated the potential changes of the Young's moduli inherent to both functionalization methods with PDA. SEM micrographs showed that the e-PTFE surface consisted of PTFE filaments that meet in knots, thus forming denser areas. Therefore, the e-PTFE material exhibits two different features, i.e., scattered threads or compact nodes (Figure 5). Mechanical properties of the raw e-PTFE, the PDA-impregnated biomaterial, and the PDA-coated surface were characterized by colloidal probe AFM (Talon et al., 2019). The histogram of the Young's moduli of the e-PTFE raw material discloses a bimodal distribution (Figure 6A) of the stiffness as expected according to the described heterogeneity of the material (Figure 5). AFM analyses carried out on the impregnated e-PTFE also revealed a bimodal distribution of the stiffness, similar to that of the raw biomaterial but showing a very slight increase of the stiffness value (Figure 6B). Remarkably, the deposition of a thin PDA film by Langmuir-Schaeffer transfer, although hiding the fibrillar architecture of the e-PTFE material (Figures 2E,F), does not alter the bimodal distribution of the stiffness (Figure 6C). Therefore, one can expect that cellular responses will not be impacted by a change of mechanical factors.

**Figure 7:**
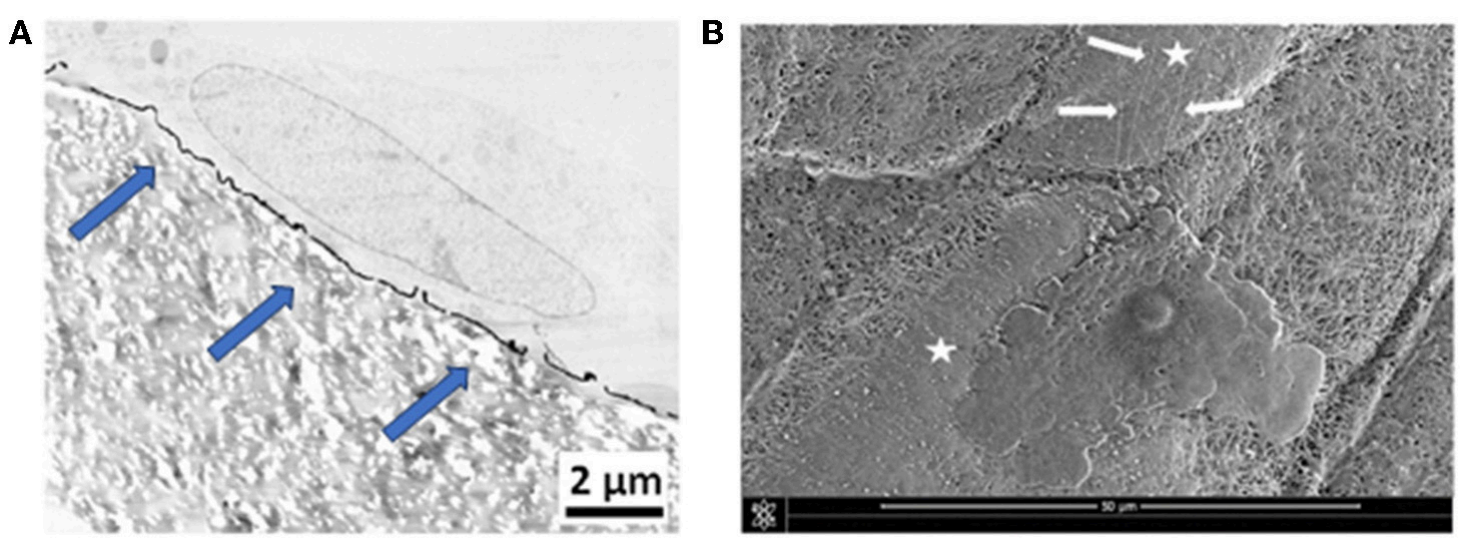
**(A)** TEM micrograph showing a primary fibroblast at the surface of PDA-coated e-PTFE. Scale bar = 2 μm. Many focal contacts of the cultured cells are evident at the surface of the thin PDA coating (blue arrows). Reproduced from Talon et al. (2019) with authorization. **(B)** ESEM micrograph showing a Wharton's jelly stem cell spread onto PDA-coated e-PTFE. Cytoplasmic projections (white arrows) are oriented toward the PDA coated area. Scale bar = 50 μm. White stars mark PDA-coated areas.

The cellular behavior of primary fibroblasts and Wharton's jelly stem cells was investigated on the PDA-impregnated and PDA-coated biomaterials, by means of electron microscopy. Results show a close contact between the cells and the biomaterials. Environmental scanning electron microscopy (ESEM) observations showed a regular spread of the cells onto PDA-impregnated e-PTFE. TEM micrographs from fibroblasts grown onto the PDA thin coating disclosed similar results. Nevertheless, cells onto the PDA coating exhibited more focal contacts. In general, the observations revealed a very close contact between the fibroblast layer and the coated e-PTFE (Figure 7A). The numerous focal contacts exhibited by the cultured cells emphasize the cell attachment to the thin PDA-coating (Figure 7A). It is interesting to note that when the cells were put onto a substrate which was imperfectly covered by the PDA layer, they grew preferentially over the PDA-coated areas and sent filopodia toward the PDA layer (Figure 7B).

The precedent findings reveal that the mechanical properties of e-PTFE at the microscopic level are not modified by both PDA treatments. Cells spread onto both PDA functionalized biomaterials. Nevertheless, it must be pointed out that microscopic observations disclose numerous focal cell contacts, evidencing cell attachment, and filopodia particularly onto the thin PDA film. This is an important finding because, filopodia are involved in cell migration.

A natural perspective of the findings described herein is to test the biological response of such membranes *in vivo*. Excellent biological responses and durability of the PDA modified prostheses are expected owing to well-known *in vivo* biocompatibility of the adhesive catecholamine based coating (Liu et al., 2014).

## Conclusions

This review underlines that PDA functionalization is a smart method to modify the surface of e-PTFE in a face specific manner. The described PDA nano-sized coating offers an easy way to modify only one side of implants used for CDH repair. As in the majority of referral centers, e-PTFE prostheses continue to be favored for the surgical repair of CDH. The observed cellular behaviors emphasize the interest of this functionalization method to optimize surgical implantation of e-PTFE to become an almost perennial surgical solution to address this important health problem. Of course, after the already performed *in vitro* evaluations, *in vivo* experiments in rats need to be done before possible clinical use of e-PTFE Janus like prostheses.

## Author Contributions

IT and AS performed the experimental work and wrote the part devoted to materials used in the repair of CDH. VB wrote the part devoted to PDA functionalization and the experimental results. JH was the project initiator and contributed to the paper writing.

### Conflict of Interest Statement

The authors declare that the research was conducted in the absence of any commercial or financial relationships that could be construed as a potential conflict of interest.
